# Modulatory effects of GLT-1 enhancer, MC-100093, on glutamate uptake and associated signaling pathways in female and male alcohol preferring rats exposed to ethanol

**DOI:** 10.1093/ijnp/pyaf075

**Published:** 2025-10-07

**Authors:** Ahmed Alotaibi, Khokon Kanti Bhowmik, Woonyen Wong, Adil Shareef Mohammed, Magid Abou-Gharbia, Wayne Childers, Youssef Sari

**Affiliations:** Department of Pharmacology and Experimental Therapeutics, College of Pharmacy and Pharmaceutical Sciences, University of Toledo, Toledo, OH, United States; Department of Pharmacology and Experimental Therapeutics, College of Pharmacy and Pharmaceutical Sciences, University of Toledo, Toledo, OH, United States; Department of Pharmacology and Experimental Therapeutics, College of Pharmacy and Pharmaceutical Sciences, University of Toledo, Toledo, OH, United States; Department of Pharmaceutical Sciences, Temple University School of Pharmacy, Philadelphia, PA, United States; Department of Pharmaceutical Sciences, Temple University School of Pharmacy, Philadelphia, PA, United States; Department of Pharmaceutical Sciences, Temple University School of Pharmacy, Philadelphia, PA, United States; Department of Pharmacology and Experimental Therapeutics, College of Pharmacy and Pharmaceutical Sciences, University of Toledo, Toledo, OH, United States

**Keywords:** nucleus accumbens, glutamate, GLT-1, xCT, MC-100093

## Abstract

**Background:**

Ethanol consumption disrupts glutamate homeostasis in several brain regions. The uptake of extracellular glutamate is regulated in the majority by the astrocytic glutamate transporter 1 (GLT-1), and cystine-glutamate exchanger (xCT) contributes to this regulatory effect. Chronic ethanol consumption is well known to downregulate GLT-1 expression in several reward brain regions, including the nucleus accumbens (NAc).

**Objectives:**

Recently, we reported that a novel beta-lactam, MC-100093, attenuated ethanol consumption and normalized the expression of GLT-1 in the subregions of the NAc. Based on these findings, we aimed in this study to determine the dose-dependent effect of MC-100093 in attenuating ethanol consumption and whether this attenuating effect is associated with restoration of glutamate uptake. In addition, we focused on whether the effects of MC-100093 on GLT-1 are mediated through the mammalian target of rapamycin (mTOR), protein kinase B (Akt), and nuclear factor-kappa B (NF-κB) signaling pathways.

**Methods:**

Male and female alcohol-preferring (P) rats are grouped into 4 groups. Other than control groups all the 3 groups had free access to ethanol (15% and 30% v/v), and water for 5 weeks. On week 6, rats received intraperitoneal injection (i.p.) of MC-100093 at a dosage of 100 or 150 mg/kg, or saline, for 5 days. The Na^+^ dependent and Na^+^ independent glutamate uptake is measured by radioactive glutamate uptake assay. The expression of GLT-1, xCT, mTOR, phospho-Akt (p-Akt), kappa light polypeptide gene enhancer in B-cells inhibitor, alpha (IkBa), and NF-κB are determined by Western blot analysis.

**Results:**

MC-100093 treatment reduced ethanol drinking in male and female P rats. MC-100093 was associated with an increase in Na^+^-dependent and Na^+^-independent glutamate uptake. Furthermore, MC-100093 treatment attenuated ethanol-induced decrease in GLT-1, xCT, NF-κB, and p-Akt expression in the NAc.

**Conclusions:**

These findings demonstrate that MC-100093 attenuated ethanol consumption and regulated glutamate uptake through normalizing GLT-1 expression.

Significance Statement This study reports the preclinical testing of novel drug, MC-100093, for the attenuation of ethanol intake. The pharmacological aspects of this drug have been shown in this study, which include the pharmacodynamic of the drug in target proteins and the modulatory effects in the uptake of neurotransmitter such as glutamate. MC-100093 treatment resulted in reduced ethanol drinking in male and female P rats. MC-100093 was associated with an increase in Na^+^-dependent and Na^+^- independent glutamate uptake. Furthermore, MC-100093 treatment attenuated ethanol-induced decrease in GLT-1, xCT, NF-κB, and p-Akt expression in the nucleus accumbens as reward brain region. Thus, the findings demonstrate that MC-100093 attenuated ethanol consumption and regulated glutamate uptake through normalizing GLT-1 expression.

## INTRODUCTION

Drug dependence has been associated in part with changes in glutamate homeostasis in central brain reward regions, including the nucleus accumbens (NAc), prefrontal cortex (PFC), and ventral tegmental area.^1^^,^^2^ The NAc receives glutamatergic inputs from the PFC, amygdala, and hippocampus, mediating drug reinforcement and drug-seeking behaviors.^1^^,^^3^ Several studies have demonstrated the involvement of glutamatergic neurotransmission in ethanol-drinking behavior.^4^^,^^5^ Disruption of glutamate homeostasis can lead to increased extracellular glutamate concentrations in the synaptic cleft, resulting in altered reward processing, relapse, and neurotoxicity.^6–8^ Glutamate transporter, specifically glutamate transporter-1 (GLT-1), a sodium (Na^+^)-dependent excitatory amino acid transporter, is responsible for the uptake of more than 90% of total extracellular glutamate.^9^ In addition, other transporter, such as cystine-glutamate exchanger (xCT), a Na^+^-independent transporter, plays an essential role in maintaining normal extracellular glutamate concentrations in reward-related brain regions.^9–14^ It is important to note that chronic exposure to ethanol and other drugs of abuse, such as cocaine and opioids, has been associated with downregulation of GLT-1 and xCT expression in the NAc,^15–19^ and dysregulation in glutamate uptake.^5^^,^^15^^,^^20^

Previous studies have shown that ceftriaxone, a beta-lactam antibiotic, effectively reduced ethanol consumption in animal models.^17^^,^^19–21^ This behavioral effect is partly attributed to the upregulation of GLT-1 expression in the NAc.^20^^,^^22^ In addition, studies from ours and others showed that increased GLT-1 expression induced by ceftriaxone is associated with reduction in cue-induced cocaine-seeking behavior and attenuation of the effect of hydrocodone overdose.^23–25^ Recently, our laboratory reported that a novel non-antibiotic beta-lactam, MC-100093, showed similar efficacy to ceftriaxone in reducing ethanol intake in male and female alcohol-preferring (P) rats. This effect is partially due to the upregulation of GLT-1 and xCT expression in the subregions of the NAc and PFC.^19^

The exact molecular mechanism by which MC-100093 and other beta-lactams upregulate or normalize GLT-1 expression has yet to be determined. Emerging evidence suggests that the protein kinase B (Akt), the mammalian target of rapamycin (mTOR), the nuclear factor-kappa B (NF-κB), and kappa light polypeptide gene enhancer in B-cells inhibitor, alpha (IkBa) signaling pathways play a role in regulating GLT-1 expression.^26^^,^^27^ Previous study from our laboratory reported that upregulation of GLT-1 following ceftriaxone treatment involved the NF-κB and Akt signaling pathways.^28^ Therefore, these signaling pathways may serve as a potential mechanism of action for MC-100093 in promoting GLT-1 upregulation.

In the present study, we investigated the effect of MC-100093 on restoring glutamate transporters and glutamate uptake following chronic ethanol consumption. We also determined whether the mTOR, Akt, and NF-κB signaling pathways are involved in MC-100093 effect on upregulating GLT-1 in the NAc of male and female P rats following chronic ethanol consumption. Furthermore, we examined the effectiveness of two different doses of MC-100093 (100 and 150 mg/kg, i.p.) in attenuating ethanol consumption in both male and female P rats.

## MATERIALS AND METHODS

### Animals and Ethanol Consumption Paradigm

**Figure 1:**
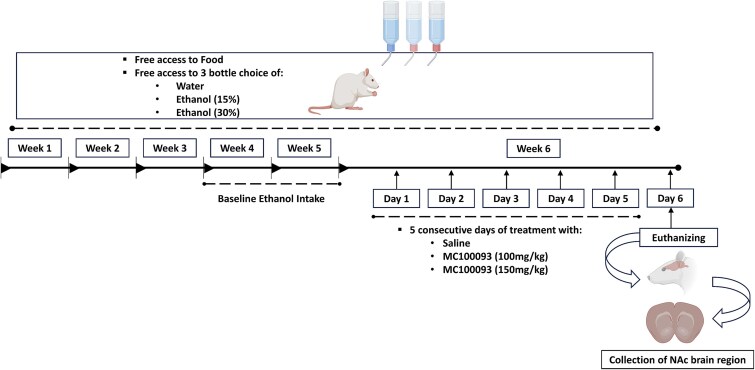
Timeline representing experimental design for ethanol drinking and drug treatments.

Male and female adult P rats were received from Indiana University School of Medicine, Indianapolis, and were housed in the Department of Laboratory Animal Resources (DLAR), The University of Toledo. Rats had free access to food and water throughout the experimental procedures. Rats were separated into four experimental groups (*n* = 5): (1) ethanol naïve group (Naïve) was only exposed to water and received saline (i.p.); (2) ethanol group (EtOH) received saline (i.p.); (3) ethanol MC-100093 group received 100 mg/kg (i.p.) of MC-100093; and (4) ethanol MC-100093 group received 150 mg/kg (i.p.) of MC-100093. Based on our power analysis, 5 animals per group were sufficient to detect a significant difference between the treatments at a 0.05 level of significant with an 80% power level.^19^^,^^29^ Rats with an average ethanol intake less than 4 g/kg/day were excluded from the study, and rats drinking more than 4 g/kg showed continuous dependence on ethanol throughout the end of the study.^17^^,^^24^^,^^30^ Ethanol groups had continuous access to free choice water and ethanol (15% and 30%, v/v) concurrently for 5 weeks. Ethanol consumption was calculated as grams per kilogram of body weight per day (g/kg/day) based on average consumption of 15% and 30% (v/v) ethanol. The baseline was calculated as the average measurements derived from the last two weeks of ethanol and water intake prior to i.p. injections of MC-100093 and saline. On week 6, rats were treated with 100 or 150 mg/kg of MC-100093 or saline as a vehicle once a day for 5 consecutive days. Ethanol preference was calculated as (ethanol intake/total fluid intake) ^*^100. Ethanol, water intake, and rat body weight were measured daily during the treatment phase of ethanol intake (Figure 1).

A separate cohort of P rats was added to the study to determine glutamate uptake using radioactive glutamate uptake assay. Male and female P rats were exposed to the ethanol-drinking paradigm as performed above. Rats were grouped into 3 groups: (1) ethanol naïve group treated with saline (i.p.), (2) ethanol group treated with saline (i.p.), and (3) ethanol group treated with MC-100093 at 100 mg/kg (i.p.) for 5 days (*n* = 5). All animal experiments were approved by The Institutional Animal Care and Use Committee at The University of Toledo (IACUC protocol #400160-UT).

### Brain Tissue Harvesting

Rats were euthanized by CO_2_ inhalation and rapidly decapitated with guillotine 24 h after the last i.p. injections of either saline or MC-100093. Brains were dissected, immediately frozen on dry ice, and stored at −80°C. The target brain region, NAc, was micro-punched using surgical blades following stereotaxic coordinates^31^ under cryostat maintained at −20°C.

### Western Blot Assay

Western blot assay was used to detect the expression of the following in the NAc: GLT-1, xCT, phospho-Akt (p-Akt), total-Akt (Akt), mTOR, NF-κB, IkBα, Lamin, and GAPDH. The NAc was homogenized, and the cytoplasmic and nuclear fractions were obtained as previously described in previous study from our laboratory.^32^ Briefly, extracted NAc samples were homogenized in buffer A containing the following: 10 mM HEPES-KOH, 1.5 mM MgCl_2_, 10 mM KCl, 1 mM dithiothreitol (DDT), 1 mM phenyl methyl sulfonyl fluoride (PMSF), and 10 uL of protease inhibitor/ml. The homogenized samples were incubated at 4°C for 10 min, and then 0.1% Nonidet P40 was added, and samples were incubated for another 2 min at 4°C. The samples were then centrifuged at 16100*g* for 15 min at 4°C. The resulting supernatant was removed, and 50 mM NaF, 10 mM Na-vanadate, and 0.1 mM Na-pyrophosphate were added to the final concentrations to obtain the cytosolic fraction. The remaining pellet was re-suspended in buffer B containing the following: 20 mM HEPES-KOH; 1.5 mM MgCl2; 25% glycerol; 420 mM NaCl; 1 mM DDT; 1 mM PMSF; 0.2 mM EDTA; 50 mM NaF; 10 mM Na vanadate; 0.1 mM Na pyrophosphate; and 10 μl of protease inhibitor and incubated in ice for 30 min. The supernatant was collected as the nuclear fraction following centrifugation of the samples at 16100*g* for 15 min at 4°C.

Total protein in each sample was quantified using a protein assay (Bio-Rad, USA). Equal concentration of protein samples was mixed with 5x Laemmli loading dye and separated into acrylamide gel (10%–20%) in Laemmli buffer. Proteins were transferred onto nitrocellulose PVDB membranes, and then blocked with 5% milk in Tris-buffered saline Tween-20 (1x TBST) for 30 min and incubated overnight at 4°C with one of the following antibodies: rabbit anti-GLT-1 antibody (1:1000; Abcam, Ab205248), rabbit anti-xCT (1:1000; Abcam, Ab175186), rabbit anti-mTOR (1:100, Cell Signaling Technology, 7C10), rabbit anti-p-Akt (1:1000, Cell Signaling Technology, D9E), rabbit anti-Akt (1:1000, Cell Signaling Technology, C67E7), rabbit anti-NF-κB (1:1000, Huabio, ER0815), mouse anti-IkBα (1:1000, Cell Signaling Technology, L35A5), rabbit anti-GAPDH antibody (1:1000, Cell Signaling Technology, D16H11), or rabbit anti-Lamin (1:1000, Huabio, ET1606-27). On the following day, membranes were washed for 30 min with 1x TBST buffer and incubated with the corresponding secondary antibody (1:4000) for 1 h. Protein density was measured using ChemiDoc imaging system (Bio-Rad, USA) following incubation with chemiluminescent reagents (Super Signal West Pico, Pierce Inc.). Band density for each detected protein was quantified and analyzed using ImageJ (version 1.53a). The mean ratios of the ethanol naïve group were used as control to normalize the ratios of animals in the ethanol saline-treated group and ethanol MC-100093-treated groups. The control ratio was set at 100, and the results from each of the treated groups were expressed as a percentage relative to the ethanol naïve group value of 100%.

### Glutamate Uptake Assay

Male and female P rats were used to evaluate the [^3^H]-glutamate uptake. Following i.p. injections of MC-100093 or saline for 5 days on day 6 of Week 6 of ethanol drinking, rats were decapitated, and brains were collected to dissect the NAc rapidly. Fractionation of the crude membrane was used to determine Na^+^-dependent and Na^+^-independent glutamate uptakes in the NAc as previously performed from our laboratory.^33^ Briefly, the freshly dissected NAc samples were homogenized in 500 μl cold 0.32 M sucrose buffer containing the following: 10 mM HEPES and 1 mM EDTA (pH 7.4), and then centrifuged at 800*g* for 10 min at 4°C. The resulting supernatants were centrifugated at 16100*g* for 20 min at 4°C to pellet down the crude membrane fractions. To determine the Na^+^-dependent glutamate uptake, the resulting pellets were suspended in Krebs–Ringer’s phosphate (KRP) buffer containing the following: 140 mM NaCl, 1.2 mM CaCl_2_, 1.2 mM KH_2_PO_4_, 5 mM HEPES, 1 mM MgCl_2_, and 10 mM glucose (pH 7.4). NaCl in KRP buffer was replaced with 140 mM choline chloride to obtain Na^+^-independent glutamate uptake. Then, 2 μCi/ml of [^3^H]-glutamate was added to initiate the uptake process in the presence of 1 μM of unlabeled glutamate in a final volume of 250 μl and incubated for 15 min at 37°C. Glutamate uptake was terminated by placing the samples on ice and were then centrifuged at 800*g* for 10 min at 4°C. The resulting pellets were further washed with ice-cold nonradioactive choline-containing KRP buffer. The pellets were solubilized with 1% SDS, and radioactivity concentrations were measured in a liquid scintillation counter. Protein concentration in each sample was quantified using a protein assay (Bio-Rad, USA), and glutamate uptake was measured as disintegrations per minute (dpm)/mg of protein/minute. Results were expressed as a ratio relative to the ethanol naïve group.

### Statistical Analysis

Two-way ANOVA followed by Tukey’s post hoc test to analyze ethanol consumption, water intake, and body weight measurements. One-way ANOVA followed by the Newman–Keuls post hoc test was performed to analyze Western Immunoblot data. Glutamate uptake was analyzed by 1-way ANOVA followed by Tukey’s post hoc. All statistical analyses data were reported as a *P <* .05 of significance. All statistical analyses were conducted using GraphPad Prism (version 10.0.1).

## RESULTS

### Effect of MC-100093 on Ethanol Drinking Behavior in Male and Female P Rats

The baseline was estimated as an average ethanol intake for the last 2 weeks prior to i.p. injections of saline or MC-100093 at two different doses (100 and 150 mg/kg). Daily average ethanol consumption (g/kg of body weight/day) was measured for 5 consecutive days starting 24 h after the first i.p. injection on day 1 and lasted until 24 h after the last i.p. injection on day 5 in P rats treated with saline and MC-100093 (100 and 150 mg/kg).

**Figure 2:**
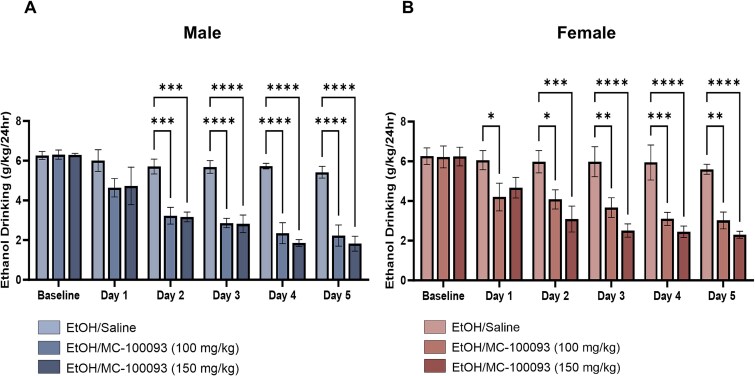
Daily average ethanol intake (g/kg/day) of male (A) and female (B) P rats treated for 5 days with MC-100093 (100 or 150 mg/kg, i.p.), or saline. Statistical analysis using two-way ANOVA followed by Tukey’s multiple comparisons test showed that the average ethanol intake was significantly reduced in groups treated with MC-100093 as compared to ethanol saline-treated group. The effect of both MC-100093 doses on ethanol drinking was profound from day 3 throughout day 5 compared to ethanol saline-group. Values are expressed as mean ± SEM (*n* = 5/group) (^*^P < .05, ^**^P < .01, ^***^P < .001, and ^****^P < .0001).

In male P rats, two-way ANOVA revealed a significant main effect of treatment and day on ethanol intake (*F*_(2, 72)_ = 60.75, *P <* .0001; *F*_(5, 72)_ = 25.43, *P <* .0001, respectively) and a significant treatment × day interaction (*F*_(10, 72)_ = 3.858, *P* = .0003). Tukey’s post hoc demonstrated a significant reduction in ethanol intake among all animals treated with MC-100093 at the doses of 100 and 150 mg/kg (*P* = .0002; *P* = .0001, respectively) on day 2. The effect of both MC-100093 doses was profound from day 3 through day 5 (*P <* .0001) compared to ethanol saline-treated group (Figure 2A). However, there was no significant difference between the 2 doses of MC-100093 on ethanol intake across all days. The effects of MC-100093 treatment in decreasing ethanol consumption are in accordance with recent studies from our laboratory.^19^^,^^34^

In female P rats, two-way ANOVA revealed a significant main effect of treatment (*F*_(2, 72)_ = 36.56, *P <* .0001), day (*F*_(5, 72)_ = 10.38, *P <* .0001) on ethanol intake, and a significant treatment × day interaction (*F*_(10, 72)_ = 2.106, *P* = .0348). Post hoc analysis using Tukey’s showed a significant reduction in ethanol intake among rats treated with MC-100093 (100 mg/kg) dose on day 1 compared to ethanol rats treated with saline (*P* = .0353). This effect was associated with a significant increase in water intake (*P* = .0252). Although MC-100093 at a dose of 150 mg/kg (i.p.) did not reveal a significant reduction in ethanol intake 24 h after the first injection, the effect of this dose was observed starting on day 2 (*P* = .0005). Similar effect was found with 100 mg/kg MC-10093 dose compared to ethanol group (*P* = .0308). On day 3, both doses of MC-100093 (100 and 150 mg/kg) were associated with a greater significant reduction in ethanol consumption (*P* = .0067; *P <* .0001, respectively). This effect was observed across days 4 and 5 of MC-100093 treatment groups compared to ethanol saline-treated group (Figure 2B).

### Effect of MC-100093 on Water Intake, Ethanol Preference, and Body Weight in Male and Female P Rats

**Figure 3:**
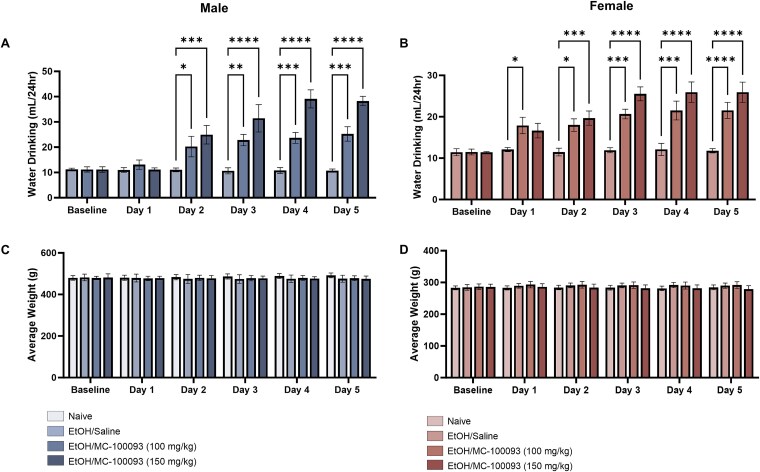
Effect of MC-100093 on daily water intake (mL/day) and average daily body weight of male and female P rats. Two-way ANOVA analyses revealed significant differences among control and treatments groups from day 2 through day 5. Tukey’s post hoc analyses revealed a significant increase in water intake from day 2 through day 5 with MC-100093 (100 and 150 mg/kg, i.p.) treatments in male (A) and female (B) P rats. Treatment with MC-100093 did not affect the body weight across the 5 treatment days as compared to saline-treated group in male (C) and female (D) P rats. Values are expressed as mean ± SEM (*n* = 5/group) (^*^P < .05, ^**^P < .01, ^***^P < .001, and ^****^P < .0001).

Two-way ANOVA revealed a significant main effect of day × treatment interaction (*F*_(10,72)_ = 7.121, *P <* .0001) and treatment main effect (*F*_(2, 72)_ = 59.22, *P >* .0001) on water consumption in male P rats (Figure 3A). Post hoc analysis demonstrated a significant increase in water intake in the MC-100093 (100 mg/kg) group on day 2 (*P* = .0229), days 3 and 4 (*P* = .0019, *P* = .0009), and day 5 (*P* = .0002) compared to ethanol group. Whereas MC-100093 (150 mg/kg) dose effect was more prominent and was associated with a significant increase in water consumption on day 2 (*P* = .0003) and days 3–5 (*P <* .0001) compared to ethanol group, and a significant increase in water intake compared to the 100 mg/kg dose on day 3 (*P* = .0361), days 4 and 5 (*P <* .001) (Figure 3A). A significant main effect of day × treatment (*F*_(10,72)_ = 3.822, *P* = .0004), and a significant main effect of treatment on water intake as well as day (*F*_(2, 72)_ = 55.74, *P <* .0001; *F*_(5, 72)_ = 13.80, *P <* .0001, respectively) was revealed by two-way ANOVA in female P rats (Figure 3B). Post hoc analysis demonstrated a significant increase in water intake in the MC-100093 (100 mg/kg) group on days 1 and 2 (*P <* .05), days 3 and 4 (*P <* .001), and day 5 (*P <* .0001) compared to ethanol group. Whereas MC-100093 (150 mg/kg) dose effect was associated with a greater significant increase in water consumption on day 2 (*P* = .001) and days 3–5 (*P <* .0001) compared to ethanol group (Figure 3B).

We further examined the effect of MC-100093 on ethanol preference. Two-way ANOVA analysis revealed a significant main effect of day × treatment on ethanol preference in male and female (*F*_(10,72)_ = 8.057, *P <* .0001; *F*_(10,72)_ = 2.818, *P* = .0053, respectively), and a significant main effect of treatment in male and female (*F*_(2, 72)_ = 92.37, *P <* .0001; *F*_(2, 72)_ = 52.22, *P <* .0001, respectively) as well as day (*F*_(5, 72)_ = 33.00 *P <* .0001; *F*_(5, 72)_ = 12.91, *P <* .0001, respectively) (Table 1). Post hoc analysis showed a significant decrease in ethanol preference in male P rats treated with both doses of MC-100093 from day 2 through day 5 (*P <* .0001). In addition, MC-100093 treatment significantly decreased ethanol preference in female P rats across all treatment days [day 1 (*P <* .05); day 2 (*P <* .001); days 3–5 (*P <* .0001)] (Table 1).

**Table 1:** Effect of MC-100093 on ethanol preference in male and female P rats.

	**Ethanol preference (%)**					
	**EtOH/Saline**		**EtOH/MC-100093 (100 mg/kg)**		**EtOH/MC-100093 (150 mg/kg)**	
**Treatment**	**Male**	**Female**	**Male**	**Female**	**Male**	**Female**
Baseline	39.02 ± 1.10	40.27 ± 2.54	38.53 ± 2.03	40.22 ± 2.44	38.96 ± 1.94	40.74 ± 3.47
Day 1	38.93 ± 2.57	38.52 ± 1.96	31.94 ± 4.37	26.95 ± 4.48^^*^^	37.54 ± 3.90	27.32 ± 3.13^^*^^
Day 2	37.99 ± 1.00	38.49 ± 4.76	20.16 ± 2.81^^****^^	20.94 ± 1.22^^***^^	17.43 ± 1.82^^***^^	20.56 ± 3.42^^***^^
Day 3	39.50 ± 2.65	39.69 ± 3.89	14.40 ± 2.02^^****^^	19.49 ± 2.71^^****^^	12.72 ± 3.21^^****^^	16.43 ± 2.77^^****^^
Day 4	38.17 ± 3.45	38.03 ± 3.94	14.80 ± 2.06^^****^^	16.60 ± 3.45^^****^^	11.47 ± 2.08^^****^^	14.72 ± 1.82^^****^^
Day 5	36.51 ± 1.45	39.07 ± 1.10	15.38 ± 0.93^^****^^	18.79 ± 2.27^^****^^	07.89 ± 0.71^^****^^	15.21 ± 3.50^^****^^

Two-way ANOVA analysis revealed significant effect of MC-100093 treatment on ethanol preference in male and female P rats. Ethanol preference was decreased in groups treated with MC-100093 in both male and female P rats. Values are expressed as mean ± SEM (*n* = 5). (^*^P < .05, ^**^P < .01, ^***^P < .001, and ^****^P < .0001).

The effects of ethanol and MC-100093 on the mean body weight are shown in (Figure 3C and D). Two-way ANOVA did not reveal a significant effect on the body weight among all groups in male and female P rats.

### Effect of MC-100093 and Ethanol Consumption on Glutamate Uptake in the NAc of Male and Female P Rats

**Figure 4:**
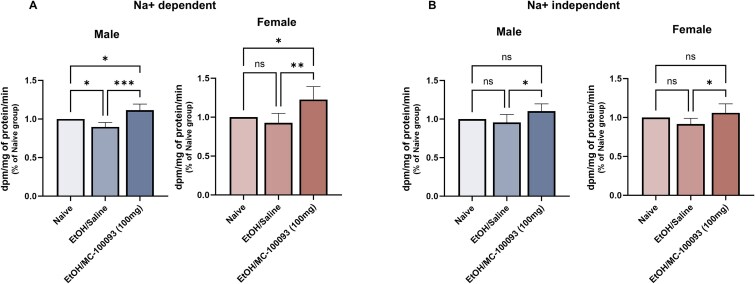
Effect of MC-100093 on Na^+^-dependent and Na^+^-independent glutamate uptake in female and male P rats exposed chronically to ethanol. Na^+^-dependent glutamate uptake was significantly increased following treatment with MC-100093 in male and female P rats compared to ethanol saline-treated group (A). There was a significant reduction in Na^+^-dependent glutamate uptake in ethanol group compared to ethanol naïve group in male P rats only (*P* < .05) (A). Treatment with MC-100093 was associated with a significant increase in the Na^+^-independent glutamate uptake in male and female P rats (B). Compared to naïve group, there was no difference in the effect of ethanol on Na^+^-independent glutamate uptake in both male and female P rats. Values are expressed as mean ± SEM (relative to 100% naïve group) (*n* = 5/group) (^*^P < .05, ^**^P < .01, ^***^P < .001, and ^****^P < .0001).

Glutamate uptake was determined using radioactive glutamate uptake assay in fresh collected NAc tissue. Analysis with one-way ANOVA showed a significant difference in the Na^+^-dependent glutamate uptake between all treatment groups in male and female (*F*_(2, 12)_ = 19.59, *P* = .0002; *F*_(2, 12)_ = 8.626, *P* = .0048, respectively). There was a significant reduction in Na^+^-dependent glutamate uptake in ethanol group compared to ethanol naïve group in male P rats only (*P <* .05) (Figure 4A). Importantly, Na^+^-dependent glutamate uptake was significantly increased following treatment with MC-100093 in male (*P <* .001) and female (*P <* .01) compared to ethanol saline-treated group (Figure 4A). Similarly, Na^+^-independent glutamate uptake was significantly different among treatment groups in male and female P rats (*F*_(2, 12)_ = 4.401, *P* = .0368; *F*_(2, 12)_ = 4.260, *P* = .0400, respectively). In a similar manner MC-100093 was associated with a significant increase in the Na^+^-independent glutamate uptake in both sexes (*P <* .05) (Figure 4B). Compared to naïve group, there was no difference in the effect of ethanol on Na^+^-independent glutamate uptake in both male and female P rats.

### Effect of MC-100093 on GLT-1 Expression in the NAc of Male and Female P Rats

**Figure 5:**
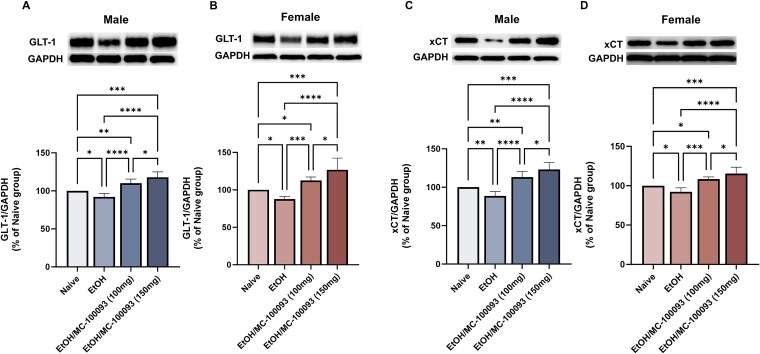
Effects of MC-100093 on the expression of GLT-1 and xCT in the NAc of male and female P rats exposed chronically to chronic ethanol. Quantitative analysis using one-way ANOVA followed by Newman–Keuls test in male P rat indicated that MC-100093 significantly upregulated GLT-1 (A) and xCT (C) expression as compared to ethanol saline-treated and ethanol naïve groups. Treatment with MC-100093 in female P rats also showed a significant upregulation of GLT-1 (B) and xCT (D) in the NAc. In addition, treatment with MC-100093 at a dose of 150 mg/kg showed a significant dose effect on upregulating GLT-1 and xCT compared to 100 mg/kg dose in male and female. Note that a significant downregulation of GLT-1 and xCT was noticed in ethanol saline-treated group compared to ethanol naïve rats in male and female. Ethanol naïve (control) group data were represented as 100%. Values are expressed as mean ± SEM (relative to 100% naïve group) (*n* = 5/group) (^*^P < .05, ^**^P < .01, ^***^P < .001, and ^****^P < .0001).

We further investigated the effects of MC-100093 on GLT-1 expression in the NAc of both male and female P rats. One-way ANOVA analyses revealed a significant main effect among ethanol naïve, ethanol saline-treated and ethanol MC-100093-treated groups in the NAc of male P rats (*F*_(3, 16)_ = 24.27, *P <* .0001) (Figure 5A), and female P rats (*F*_(3, 16)_ = 20, *P <* .0001) (Figure 5B). Newman–Keuls *post-hoc* test showed a significant increase in GLT-1 expression in the ethanol MC-100093-treated groups compared to the ethanol saline-treated group (*P <* .0001). In addition, treatment with MC-100093 (100 mg/kg, i.p.) showed a significant increase GLT-1 expression compared to ethanol naïve group (*P <* .01), while MC-100093 at a dose of 150 mg/kg (i.p.) was associated with profound upregulation of GLT-1 compared to ethanol naïve group (*P <* .001) and MC-100093 (100 mg/kg, i.p) group (*P <* .05). Similar effect was observed in female P rats as such MC-100093 (150 mg/kg, i.p.) increased the expression of GLT-1 as compared to ethanol naïve and ethanol saline-treated groups (*P <* .001; *P <* .0001, respectively). Although, the dose of MC-100093 at 100 mg/kg (i.p.) was associated with upregulation of GLT-1 compared to ethanol naïve and ethanol saline-treated group (*P <* .05; *P <* .001, respectively), the effect of MC-100093 at dose of 150 mg/kg (i.p.) was greater than the dose of MC-100093 at 100 mg/kg (i.p.) in the NAc of both male and female P rats (*P <* .05). Importantly, a significant downregulation of GLT-1 expression was observed in the ethanol saline-treated group compared to ethanol naïve group in the NAc of male and female P rats (*P <* .05).

### Effect of MC-100093 on xCT Expression in the NAc of Male and Female P Rats

We further examined whether chronic ethanol consumption and MC-100093 affect other glutamatergic transporters such as xCT. One-way ANOVA analysis revealed a significant difference between all treated male groups (*F*_(3, 16)_ = 30.92, *P <* .0001) (Figure 5C), and female groups (*F*_(3, 16)_ = 20.04, *P <* .0001) (Figure 5D). MC-100093 (100 mg/kg, i.p.) induced a significant upregulation in xCT expression in the NAc of male and female P rats as compared to ethanol saline-treated group (*P <* .0001). A higher dose of MC-100093 (150 mg/kg, i.p.) was associated with a greater increase in the expression of xCT in both genders compared to ethanol naïve and ethanol saline-treated groups (*P <* .0001). Importantly, MC-100093 effect on xCT demonstrated a dose-dependent effect, displaying a significant difference between the two doses in male and female (*P <* .05). Relatively, a significant downregulation of xCT was observed in ethanol saline-treated groups as compared to ethanol naïve groups of male and female P rats (*P <* .01; *P <* .05, respectively).

### Effect of MC-100093 on mTOR and Phospho-Akt (p-Akt) Expression in the NAc of Male and Female P Rats

**Figure 6:**
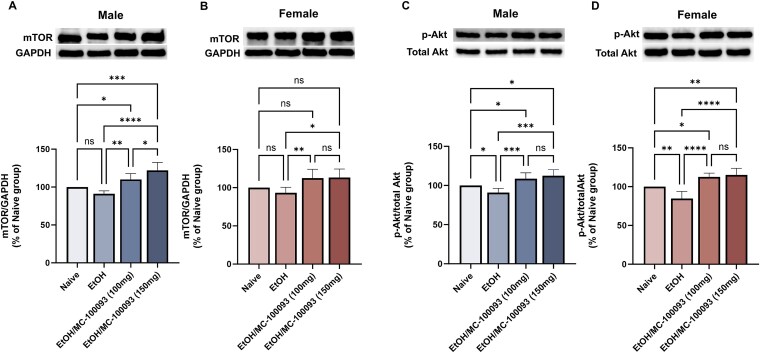
Effects of MC-100093 on the expression of mTOR and p-Akt in the NAc of male and female P rats exposed chronically to ethanol. In male P rats, one-way ANOVA followed by Newman–Keuls test revealed that MC-100093 significantly increased the expression of mTOR (A) and the phosphorylation of Akt (C) in the NAc as compared to ethanol. In female P rats, treatment with MC-100093 showed a significant increase in mTOR expression (B) and in the phosphorylation of Akt (D) in the NAc. Notably, a dose of 150 mg/kg of MC-100093 showed a significant increase in the expression of mTOR in male P rats only as compared to the 100 mg/kg dose. There was no significant difference between the two doses of MC-100093 in the phosphorylation of Akt. However, a significant reduction in the phosphorylation of Akt was observed in the ethanol saline-treated groups compared to ethanol naïve rats. Ethanol naïve (control) group data were represented as 100%. Values are expressed as mean ± SEM (relative to 100% naïve group) (*n* = 5/group) (^*^P < .05, ^**^P < .01, ^***^P < .001, and ^****^P < .0001).

We explored the involvement of targeted signaling pathways to determine the potential mechanism of action of MC-100093 on the upregulation of GLT-1 in the NAc. We focused on mTOR and Akt signaling pathways, which have been shown to be implicated in the regulation of GLT-1 expression.^35^^,^^36^ Across all groups, there was a significant main effect on the protein expression of mTOR in the NAc of male and female P rats [*F*_(3, 16)_ = 18.92, *P <* .0001; *F*_(3, 16)_ = 6.03, *P* = .006, respectively] and the expression of p-Akt in the NAc of male and female P rats (*F*_(3, 16)_ = 12.54, *P* = .0002; *F*_(3, 16)_ = 21.34, *P <* .0001, respectively). We revealed a significant increase in the expression of mTOR in the NAc of male P rats following treatment with MC-100093 at doses of 100 and 150 mg/kg (i.p.) (*P <* .01; *P <* .0001, respectively) (Figure 6A). In addition, we observed similar effect with the two doses of MC-100093 on mTOR expression in female P rats, 100 mg/kg (i.p.) (*P <* .01) and 150 mg/kg (i.p.) (*P <* .05) (Figure 6B). Importantly, chronic ethanol consumption reduced the phosphorylation of Akt in the NAc of male and female P rats (*P <* .05; *P <* .01, respectively). This effect was attenuated by MC-100093 treatment, which resulted in the upregulation of phosphorylated Akt in the NAc of both male and female (*P <* .001; *P <* .0001, respectively) (Figure 6C and D).

### Effect of MC-100093 on IkBa and NF-κB-p65 Expression in the NAc of Male and Female P Rats

**Figure 7:**
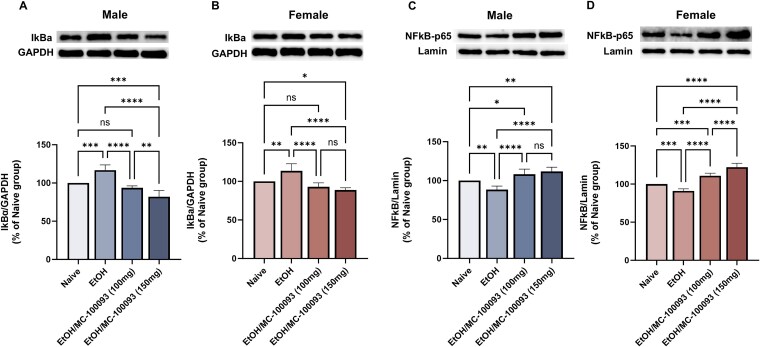
Effects of MC-100093 on the expression of IkBa and NF-κB in the NAc of male and female P rats exposed chronically to ethanol. (A) In male P rats, one-way ANOVA followed by Newman–Keuls test revealed that MC-100093 significantly decreased the level of IkBa expression in the NAc compared to ethanol saline-treated group. (B) In female P rats, treatment with MC-100093 also showed a significant downregulation of IkBa in the NAc. However, a significant increase in the expression of IkBa was seen in the NAc of ethanol saline-treated group compared to ethanol naïve rats in both sexes. In addition, a dose of 150 mg/kg of MC-100093 showed a significant decrease in the expression of IkBa as compared to the 100 mg/kg dose in male P rats only. Notably, MC-100093 significantly increased the nuclear levels of NF-κB in male (C) and female (D) P rats as compared to ethanol saline-treated and ethanol naïve groups. A dose of 150 mg/kg of MC-100093 showed a significant increase in the expression of NF-κB as compared to the 100 mg/kg dose in female P rats only. However, a significant downregulation of NF-κB was observed in ethanol saline-treated group compared to ethanol naïve male and female rats. Ethanol naïve (control) group data were represented as 100%. Values are expressed as mean ± SEM (relative to 100% naïve group) (*n* = 5/group) (^*^P < .05, ^**^P < .01, ^***^P < .001, and ^****^P < .0001).

We next investigated other signaling pathways that have potential role in the upregulatory effects of MC-100093 on GLT-1. We determined the protein expression of IkBa in the cytoplasmic fraction in the NAc. Statistical analyses showed a significant main effect on IkBa expression [*F*_(3, 16)_ = 34.48, *P <* .0001] among all groups in male P rats (Figure 7A). Similarly in female, a significant main effect among all treatment groups on IkBa [*F*_(3, 16)_ = 19.67, *P <* .0001] (Figure 7B). MC-100093 treatment was associated with a significant decrease in the expression of cytoplasmic IkBa in the NAc of male and female P rats compared to saline-treated ethanol group (*P <* .0001). Accordingly, the decrease in the IkBa expression is suggested to mediate the nuclear translocation and activation of NF-κB.^37^ Therefore, we further investigated the protein expression of NF-κB in the nuclear fraction. One-way ANOVA revealed a significant main effect on NF-κB expression in the nuclear fraction in the NAc of male [*F*_(3, 16)_ = 23.15, *P <* .0001] (Figure 7C) and female P rats [*F*_(3, 16)_ = 77.94, *P <* .0001] (Figure 7D) P rats. Notably, treatment with MC-100093 (100 and 150 mg/kg, i.p.) showed a significant increase in the expression of nuclear NF-κB in the NAc of male and female P rats compared to saline-treated group (*P <* .0001).

## DISCUSSION

In this study, we report that treatment with MC-100093 at doses of 100 and 150 mg/kg (i.p.) for 5 consecutive days reduced ethanol drinking in male and female P rats. Importantly, we suggest here that the effect of MC-100093 in decreasing ethanol drinking was partly associated with the attenuation of chronic ethanol-induced downregulation of GLT-1 and decrease in the Na^+^-dependent glutamate uptake in the NAc of male and female P rats. These results align with previous finding where we demonstrated the effectiveness of MC-100093 in attenuating ethanol consumption in male and female P rats.^19^ Although, in the previous study, MC-100093 treatment showed a significant decrease in ethanol consumption from day 1,^19^ in this study, MC-100093 treatment was effective in reducing ethanol drinking from day 2. There are several factors that could hinder the drug effects on day 1 as compared to previous study, and this might include differences in animal ages and the seasonal period of exposing animals to ethanol. Furthermore, the probable acute treatment effect of MC-100093 can be expected as a possible result of direct activation of GLT-1 and probably xCT as it has been observed with ceftriaxone, beta-lactam antibiotic. It is important to note that study from our lab showed that ceftriaxone can upregulate GLT-1 in the PFC after 48 h of treatment.^28^ Further studies are warranted to determine whether MC-100093 has a direct activating effect on GLT-1 after 24 h of drug treatment using microdialysis for measurement of glutamate uptake. Moreover, there was no significant difference in the effects of MC-100093 at 100 mg/kg (i.p.) and 150 mg/kg (i.p.) doses on reducing ethanol intake in both sexes across all treatment days. The selection of 150 mg/kg dose was based on modest escalation from the 100 mg/kg dose and was intended to explore dose-dependent effect on ethanol drinking. Nevertheless, treatment with MC-100093 was associated with an increase in the water intake in both male and female P rats compared to the saline-treated group. Furthermore, treatment with MC-100093 decreased the ethanol preference. This decrease in ethanol consumption was associated with an increase in water consumption and a decrease in ethanol preference.

Moreover, we demonstrated that MC-100093 did not affect the body weight of rats. A previous study reported that treatment with MC-100093 did not affect sucrose intake,^38^^,^^39^ and no effect was found on body weight of rats^38^ and mice.^39^ These findings are consistent with previous studies reporting that treatment with MC-100093 or ceftriaxone had no effect on body weight and sucrose intake.^17^^,^^34^^,^^38^^,^^40^ Thus, these findings highlight the specificity of MC-100093 in reducing ethanol drinking without impacting the body weight of the animals.

Chronic ethanol consumption has been linked to the dysregulation of the glutamatergic pathways in the mesolimbic reward system, specifically in the NAc.^41^^,^^42^ In addition, chronic ethanol consumption was found to downregulate the expression of key glutamate transporters, including GLT-1 and xCT, responsible for the regulation of the majority of extracellular glutamate concentrations in the NAc.^19^^,^^22^^,^^28^ Therefore, this can lead to the disruption in the function of these transporters and increase in extracellular glutamate concentrations with chronic ethanol consumption. It is important to note that studies demonstrated that exposure to ethanol and other drugs of abuse, including cocaine, can lead to reduction in glutamate uptake.^5^^,^^15^^,^^20^ Thus, in the present study, we investigated whether the effect of MC-100093 in attenuating ethanol consumption is partially associated with the normalization of glutamate uptake and associated glutamate homeostasis in the NAc of P rats. Our results showed a significant reduction of GLT-1 and xCT after chronic exposure to ethanol in the NAc of male and female P rats, consistent with a decrease in the Na^+^-dependent glutamate uptake. Treatment with MC-100093 for 5 days increased the expression of both transporters in the NAc of male and female P rats. Importantly, the apparent effect of MC-100093 on increasing the protein expression of both transporters was associated with increased Na^+^-dependent and Na^+^-independent glutamate uptake in the NAc of both male and female P rats. In accordance, treatment with ceftriaxone was also found to be associated with an increase in glutamate uptake in cocaine animal models.^15^^,^^43^ Previous studies from our lab have shown that chronic ethanol increased extracellular glutamate concentrations in the NAc.^20^ This increase is suggested to lead to activation of medium spiny neurons (MSNs) and alteration of MSN excitability, resulting in increased ethanol seeking behavior.^44^^,^^45^ Importantly, modulation of the NAc neuronal activity can affect ethanol intake.^46^ Therefore, pharmacological manipulations that restore glutamate homeostasis through the upregulation of GLT-1 or xCT are expected to attenuate NAc neuronal hyperactivity by reducing extracellular glutamate spillover and excitotoxicity. Thus, the effect of MC-100093 in reducing ethanol intake may lead to the normalization of NAc neuronal activity through normalizing the expression of GLT-1 as well as xCT. Future studies are warranted to investigate the effect of MC-100093 on NAc neuronal activity using electrophysiological and in vivo glutamate signal recordings. Furthermore, ceftriaxone was found to decrease extracellular glutamate concentrations in the NAc,^20^ which might also indicate an increase in the glutamate uptake. Therefore, restoring the normal function of both transporters and maintaining glutamate homeostasis might be associated in part with the effect of MC-100093 on attenuating ethanol intake.

In this study, statistical analysis didn’t show any significant difference between 100 and 150 mg/kg of MC-100093 in terms of ethanol drinking. These findings suggest that 100 mg/kg was effective to attenuate chronic ethanol-induced downregulation of GLT-1 expression, and consequently reduced ethanol intake. Furthermore, chronic ethanol consumption reduced GLT-1 expression in selected brain regions, and MC-100093 at 100 mg/kg was at the optimal dose to attenuate this downregulation; and increasing the dose of MC-100093 to 150 mg/kg may as well attenuate this downregulation of GLT-1 without significantly affecting the behavioral outcomes of ethanol intake compared to the 100 mg/kg. This could be attributed to pre/postsynaptic glutamate receptors adaptation, in which further upregulation of GLT-1 by the higher dose of MC-100093 results in further reduction in glutamate levels beyond the basal glutamate levels resulting in neuronal adaptations hindering the drug effect on drinking behaviors. Similar effect was observed in cocaine-dependent model, in which higher dose of MC-100093 did not translate into a larger behavioral effect on cocaine reinstatement.^38^ Therefore, a dose-dependent effect resulted in the difference between MC-100093 100 mg/kg (i.p.) and 150 mg/kg (i.p.) in upregulating xCT and GLT-1 expression in the NAc.

**Figure 8:**
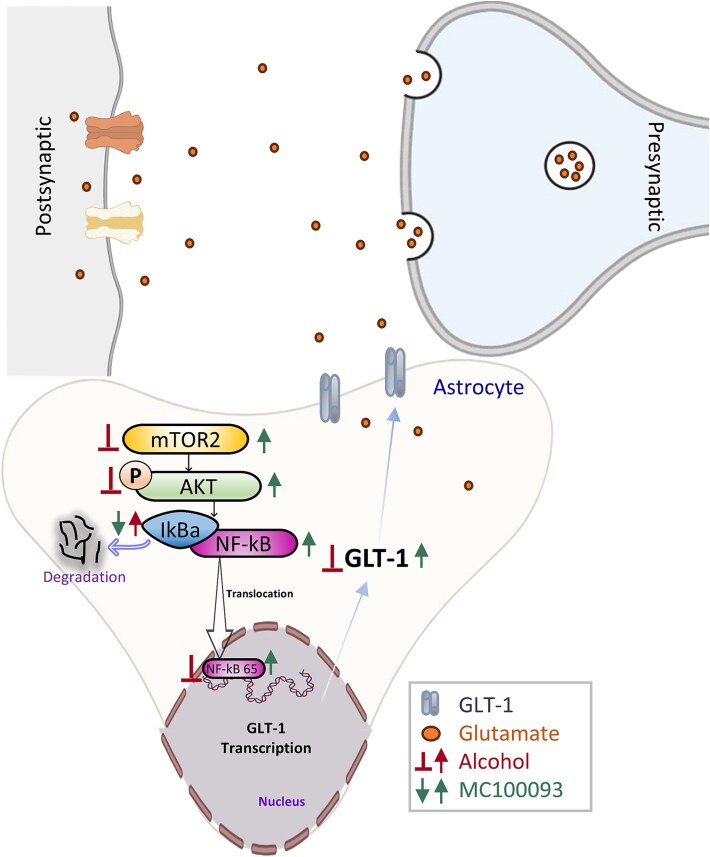
Schematic diagram represents the molecular mechanism of MC-100093 in modulating glutamate transporter 1 (GLT-1) signaling pathway following chronic ethanol consumption. Chronic ethanol consumption decreases the expression of GLT-1 through decreasing the expression of mTOR and the phosphorylation of Akt as well as the translocation and activation of NF-κB. Treatment with MC-100093 reversed the effect of ethanol on p-Akt and NF-κB signaling pathways resulting in GLT-1 upregulation. The activation of mTOR2 is known to phosphorylate and activate Akt leading to the dissociation of the cytoplasmic inhibitor IkBa protein binding to the NF-κB complex. The IkBa protein is involved in proteasomal degradation, releasing NF-κB, which can result in the activation and translocation of NF-κB to the nucleus to promote expression of GLT-1 promoter.

The mechanistic pathways regulating GLT-1 expression are complex and involve several signaling cascades, including Akt, mTOR, and NF-κB.^26^^,^^27^ Thus, to evaluate the involvement of these pathways involving GLT-1 upregulation, we further explored their influence following the treatment of MC-100093. The protein kinase mTOR, a signaling pathway involved in metabolism, protein synthesis, neuronal plasticity, and growth, generates two functional complexes. The first complex is mTOR complex 1 (mTORC1), which forms after the interaction with a raptor, and the second complex interacts with the Rictor forming mTOR complex 2 (mTORC2).^47^^,^^48^ Activation of mTORC2 is known to phosphorylate and activate Akt, a key regulator of cell survival and metabolism.^49^ mTORC2 is activated following the inhibition of mTORC1, which suggests a link between mTOR and Akt.^36^ Previous studies have shown that activation of the Akt pathway promotes the upregulation of GLT-1,^28^^,^^32^^,^^50^ enhancing its uptake function. Here, we report that treatment with MC-100093 increased the expression of mTOR and, in turn, increased phosphorylation of Akt in the NAc of male and female P rats. A similar effect was observed with ceftriaxone treatment and other beta-lactam antibiotics in P rats, where there was an increase in Akt phosphorylation.^28^^,^^51^ These findings suggest that Akt and mTOR might be involved in the regulation of GLT-1 expression, indicating that they could be potential downstream targets for the effects of MC-100093 on GLT-1 modulation (Figure 8).

Among these downstream pathways, the NF-κB pathway has been shown to play a role in the transcriptional regulation of GLT-1.^26^ NF-κB can be activated by Akt, resulting in the transcriptional activation of GLT-1.^35^^,^^52^ The cytoplasmic inhibitor IkBa protein binding to the NF-κB complex goes through proteasomal degradation, releasing NF-κB, which can result in the activation and translocation of NF-κB to the nucleus to promote expression of targeted genes.^37^^,^^50^ Therefore, activation of NF-κB can induce the activation of GLT-1 promoter.^52^ In this study, chronic ethanol consumption increased the expression of IkBa, limiting the disassociation of NF-κB and blocking its translocation to the nucleus and the transcriptional upregulation of GLT-1. As a result, NF-κB expression significantly decreased in the nucleus compartment of the neuron. In contrast, treatment with MC-100093 at doses of 100 mg/kg (i.p.) and 150 mg/kg (i.p.) significantly reduced the protein expression of IkBa, leading to the nuclear translocation and activation of NF-κB. MC-100093 was also associated with a significant increase in the expression of NF-κB in the nuclear fraction, enhancing its binding to GLT-1 promoter (Figure 8). In line with these findings, ceftriaxone was shown to exert its effects in upregulating GLT-1 by inducing NF-κB translocation from the cytoplasm to the nucleus via the degradation of the cytoplasmic inhibitor IkBa.^26^^,^^28^^,^^50^ These findings support the existing body of evidence that highlights the role of the NF-κB pathway in regulating GLT-1 and the role of MC-100093 and other beta-lactams, such as ceftriaxone, in modulating this pathway as a potential mechanism for their GLT-1 upregulation.

Collectively, this study confirmed the efficacy of MC-100093 at 100 mg/kg, and further investigated the effect of extended dosage range of MC-100093 at 150 mg/kg in both sexes. Importantly, this study reports for the first time that MC-100093 attenuated the effect of chronic ethanol intake on glutamate uptake, which was associated in part with restoring the expression of GLT-1 and xCT in the NAc. In addition, this study reports for the first time that the upregulatory effect of MC-100093 might be mediated through the Akt/mTOR/NF-κB intracellular signaling pathways, which are well known to regulate GLT-1 transcription^26^^,^^53^ as it has been found with the beta-lactam antibiotic ceftriaxone.^28^ In addition, these findings suggest that the MC-100093 effect in reducing ethanol consumption in both sexes of P rats primarily occurs through the modulation of GLT-1. GLT-1 is essential in reducing the excitatory effect that results from imbalance glutamate homeostasis. A deficit in GLT-1 is associated with a reduction in glutamate uptake involving the Na^+^-dependent glutamate mechanism. We also report a non-dose-dependent effect with MC-100093 in attenuating ethanol drinking. In addition, our present study indicates that the potential molecular pathways through which MC-100093 upregulates GLT-1 include the phosphorylation of Akt and enhanced activation of NF-κB in the NAc of both male and female P rats (Figure 8). The self-administering of multiple bottles choice paradigm for 5 weeks is an established model of ethanol drinking, which is known to develop dependence to ethanol in P rats.^54^^,^^55^ This method has been widely used to assess the pharmacological effects of drugs targeting glutamate transporters, including ceftriaxone.^17^^,^^18^ Thus, the current data from the three-bottle choice experiment offer sufficient evidence of the pharmacological efficacy of MC-100093 in decreasing voluntary ethanol consumption. However, drug dependence is a complex disorder characterized by both voluntary consumption and compulsive seeking behaviors, as well as relapse. Future studies utilizing operant and compulsive alcohol models would be crucial to examine MC-100093 potential impact on the wider motivational and reward-seeking aspects of alcohol use disorder. Studies are warranted to test the long-lasting effects of MC-100093 in ethanol intake to determine the efficacy of the compound after the 5 days treatment regimen. Furthermore, studies are warranted to explore other possible mechanisms mediating MC-100093 in the upregulation of GLT-1 and the maintenance of glutamate homeostasis, such as glutamate/glutamine synthesis cycle.

## Data Availability

The data underlying this article will be shared upon reasonable request to the corresponding author.
